# Molecular cytogenetic studies in the ladybird beetle *Henosepilachna argus* Geoffroy, 1762 (Coleoptera, Coccinellidae, Epilachninae)

**DOI:** 10.3897/CompCytogen.v9i3.5263

**Published:** 2015-07-09

**Authors:** Pablo Mora, Jesús Vela, Olivia Sanllorente, Teresa Palomeque, Pedro Lorite

**Affiliations:** 1 Departamento de Biología Experimental. Universidad de Jaén. 23071 Jaén. Spain

**Keywords:** *Henosepilachna argus*, karyotype, C-banding, DAPI staining, NOR, telomeres

## Abstract

The ladybird *Henosepilachna argus* Geoffroy, 1762 has been cytogenetically studied. In addition we have conducted a review of chromosome numbers and the chromosomal system of sex determination available in the literature in species belonging to the genus *Henosepilachna* and in its closely related genus *Epilachna*. Chromosome number of *Henosepilachna argus* was 2n=18, including the sex chromosome pair, a common diploid chromosome number within the tribe Epilachnini. The study of prophase I meiotic chromosomes showed the typical Xy_p_ “parachute” bivalent as in the majority of species of Coccinellidae. C-banding and fluorescent staining with AT-specific DAPI fluorochrome dye have been carried out for the first time in H. argus. C-banding technique revealed that heterochromatic blocks are pericentromerically located and DAPI staining showed that this heterochromatin is AT rich.

Fluorescence in situ hybridizations using rDNA and the telomeric TTAGG sequence as probes have been carried out. FISH using rDNA showed that the nucleolar organizing region is located on the short arm of the X chromosome. FISH with the telomeric sequence revealed that in this species telomeres of chromosomes are composed of the pentanucleotide TTAGG repeats. This is the first study on the telomeric sequences in Coccinellidae.

## Introduction

Insects are one of the most diverse and biggest numerous groups of metazoans. This group contains almost one million of species, half a million of which are phytophagous. Most of those phytophagous insects are considered specialist feeding on one or few plant species (Schoonhoven et al. 2005). *Henosepilachna argus* Geoffroy, 1762 (Coleoptera, Coccinellidae) the bryony ladybird is a phytophagous insect (both larvae and adults) which feeds on leaves of white bryony and other Cucurbitaceous plants, including melon or watermelon crops.

The tribe Epilachnini is included in the Epilachninae subfamily (Coccinellidae, Epilachninae) (Jadwiszczak and Wegrzynowicz 2003). Within Epilachnini, one of the most problematic questions is the distinctiveness of the genera *Epilachna* and *Henosepilachna*. Although both genera have been distinguished by morphological characters (Li 1993), that question is not fully elucidated. Recently Katoh et al. (2014) by using a combined dataset of NADH dehydrogenase subunit 2 (ND2) and the 28S rDNA reconstructed the phylogeny of 46 species of Epilachnini, including 16 species of *Epilachna* and 24 species of *Henosepilachna*. The results obtained by Katoh et al. (2014) suggest that *Henosepilachna* and *Epilachna* are polyphyletic but also the existence of some well-supported clades, such as Asian *Epilachna*, American *Epilachna* and Asian and Australian *Henosepilachna*. Despite this, Katoh et al. (2014) recommended that a new phylogenetic analysis has to be done, with special careful attention to both morphological and molecular analyses with a broad taxonomic representation. Thereby the taxonomy of the species belonging to the genus *Henosepilachna* remains unclear with misidentification for some species. Sometimes it is due to the existence of intraspecific variation which causes a wide variation in the external appearance and morphological characters presented by the species of this group (Naz et al. 2012).

In this paper a karyotype analysis, C-banding and fluorescent staining with the AT-specific DAPI fluorochrome dye have been carried out for the first time in *Henosepilachna* argus. In addition we have conducted a review of chromosome numbers and the chromosomal system of sex determination available in the literature in species belonging to the genera *Henosepilachna* and *Epilachna*. Fluorescence in situ hybridizations using rDNA and (TTAGG)n as probes have also been carried out for the first time in Epilachninae. This molecular cytogenetic study could be helpful in the future for solving the problem of distinctiveness of both genera.

## Material and methods

### Chromosome preparations, C-banding and DAPI staining

Chromosome spreads were obtained from adult male gonads according to the technique described by Lorite et al. (1996a). C-banding was performed as described by Sumner (1972) with some modifications (Palomeque et al. 2005). Staining of the chromosomes with 4’-6-diamino-2-fenil-indol (DAPI) was performed according to Schweizer (1980).

### Fluorescence *in situ* hybridization

The physical mapping of 18S and 28S rDNA loci was carried out by fluorescence *in situ* hybridization (FISH). FISH was performed as described previously (Lorite et al. 2002a, Palomeque et al. 2005). A plasmid containing the *Drosophila melanogaster* Meigen, 1830 rDNA gene (pDmr.a 51#1) (Endow 1982) was used as probe. The telomeric DNA probe was generated by polymerase chain reaction (PCR) using the (TTAGG)_6_ and (TAACC)_6_ oligonucleotides as primers (Lorite et al. 2002b). Both probes were labeled with biotin-16-dUTP using the biotin labeling kit from Roche (final concentration of 2 ng/ml, 50% formamide). Fluorescence immunological detection was performed using the avidin-FITC/anti-avidin-biotin system with two rounds of amplification for the rDNA probe and four rounds of amplification for the telomeric probe. Slides were counterstained with propidium iodide and DAPI.

## Results and discussion

**Figure 1.**
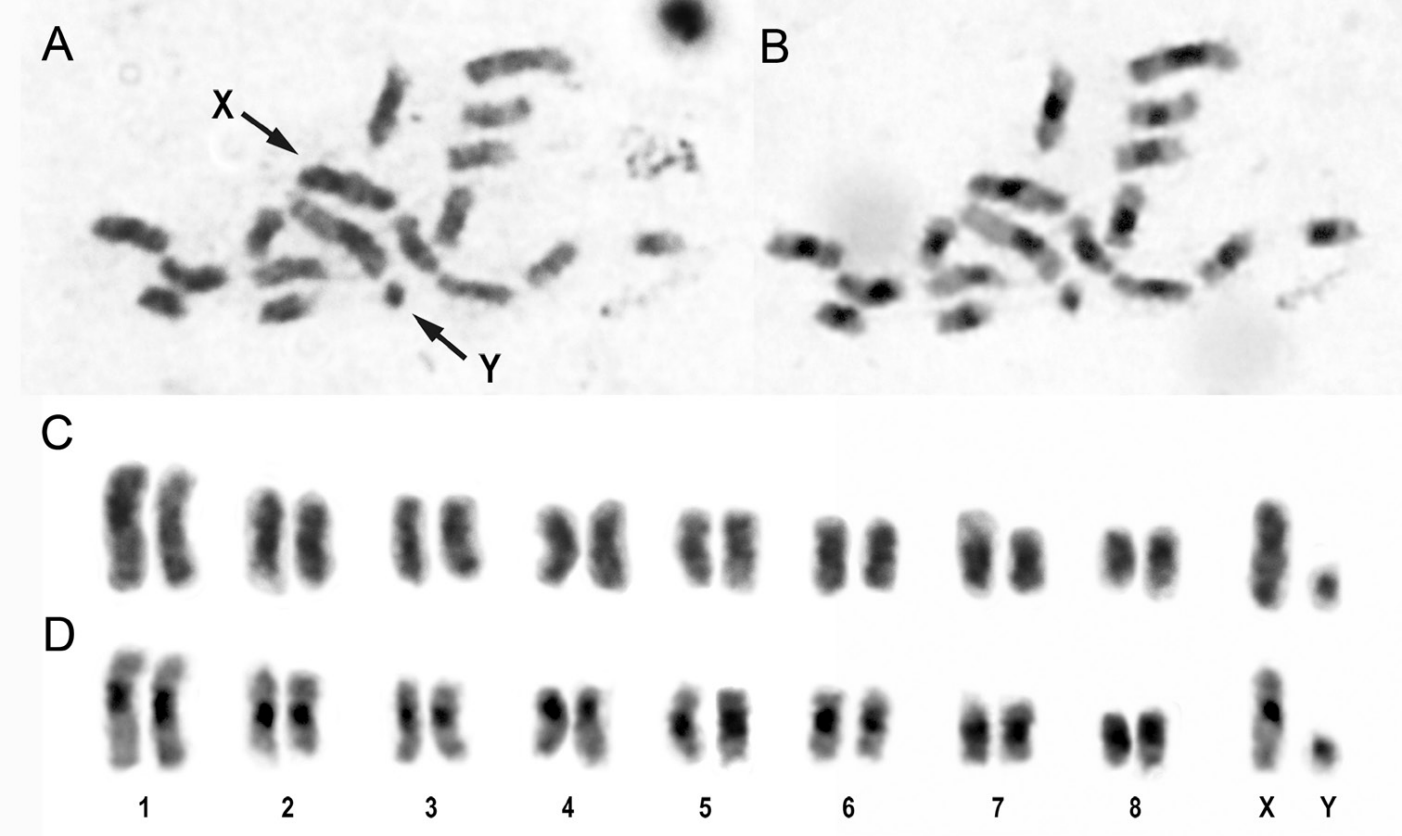
Metaphase plate and karyotype of *Henosepilachna argus* male. Giemsa staining (**A, C**) and C-banding (**B, D**). The arrows indicate the sex chromosomes (X and y).

*Henosepilachna argus* showed 8 pairs of autosomes and the sex chromosomes X and Y. The karyotype was composed of 6 pairs of metacentric (pairs 1, 2, 3, 5, 6 and 7) and 2 pairs of submetacentric autosomes (pairs 4 and 8). The chromosome X was submetacentric and the chromosome Y was minute and seems to be acrocentric (Figure 1A and C). We have conducted a review of chromosome numbers and chromosomal system of sex determination available in the literature in species belonging to the genera *Henosepilachna* and *Epilachna* (Table 1). A variable chromosome number was given for *Henosepilachna dodecastigma* Wiedemann, 1934 with a chromosome number ranging from 6 pairs of autosomes and the sex chromosome pair to 9 pairs of autosomes and the sex chromosome pair (review in Smith and Virkki 1978). However the results showed by Saha (1973) suggest that the 2n = 14 is the most probable chromosome number for this species. According to Sloggett and Honĕk (2012) the most common diploid chromosome number within Epilachnini was 18-20 (including the sex chromosome pair), as happens in *Henosepilachna argus* with a chromosome number of 2n=18.

**Table 1.** Known chromosome numbers and chromosomal system of sex determination in species belonging to the genera *Epilachna* and *Henosepilachna*.

***Epilachna***		
	2n	References
*Epilachna admirabilis* Crotch, 1874	18 Xy	Tanaka and Sasaji (1992)
*Epilachna borealis* Fabricius, 1775	18 Xy	Stevens (1906), Hoy (1918)
*Epilachna cacica* Guerín-Meneville, 1844	20 Xy	Vidal (1984)
*Epilachna dumerili* Mulsant, 1850	16 Xy	Yadav et al. (1991)
*Epilachna obscurella* Mulsant, 1850	18 Xy	Gomez and Castorena (1972)
*Epilachna paenulata* Germar, 1824	18 Xy	Drets et al. (1983)
*Epilachna varivestris* Mulsant, 1850	20 Xy	Gomez and Castorena (1972)
***Henosepilachna***		
*Henosepilachna chrysomelina* Fabricius, 1775 × *capensis* Thunberg, 1784	18	Strasburger (1936)
*Henosepilachna dodecastigma* Wiedemann, 1823	20 Xy	Lahiri and Manna (1969)
	12-14	Saha and Manna (1971)
	14 Xy	Saha (1973)
*Henosepilachna niponica* Lewis, 1896	20 Xy	Yosida (1944), Tanaka and Sasaji (1992), Tsurusaki et al. (1993)
*Henosepilachna orientalis* Zimmerman, 1936	18 XY	Agarwal (1961)
*Henosepilachna pustulosa* Kono 1937	20 Xy	Yosida (1948), Tsurusaki et al. (1993)
*Henosepilachna septima* Dieke, 1947	20 Xy	Kacker (1973)
*Henosepilachna vigintioctomaculata* Motschulsky, 1857	20 Xy	Yosida (1948), Takenouchi (1955), Tsurusaki et al. (1993)
*Henosepilachna vigintioctopunctata* Fabricius, 1775	18 Xy; XY	Bose (1948), Yosida (1948), Agarwal (1961), Yadav and Pillai (1979), Tanaka and Sasaji (1992), Kobayashi et al. (2000)
*Henosepilachna yasutomii* Katakura, 1981	20 Xy	Tsurusaki et al. (1993)

Differential chromosomal staining is able to show some specific patterns helpful to distinguish chromosomes with the same size. C-banding reveals the constitutive heterochromatin (Sumner 1972). In Coccinellidae beetles the heterochromatin is associated with pericentromeric regions and shorts arms of chromosomes (Drets et al. 1983, Maffei et al. 2004, Rozek and Holecova 2002, among others). The application of C-banding techniques in *Henosepilachna argus* showed large heterochromatic pericentromeric blocks on all chromosomes. The small chromosome Y was almost entirely heterochromatic (Figure 1B and D). Similar results have been reported for other Epilachnini although only four species have been analyzed by C-banding methods (Drets et al. 1983, Tsurusaki et al. 1993).

**Figure 2.**
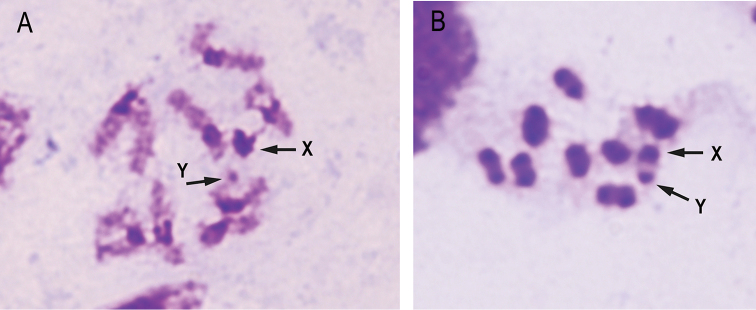
Giemsa staining of meiotic chromosomes at late pachytene (**A**) and in metaphase I (**B**). The arrows indicate the sex chromosomes (X and y).

The sex chromosomal system found in *Henosepilachna argus* is XX/Xy_p_ (Figure 2). The y chromosome was minute and for this reason it is often written with lowercase letter. When X and y chromosomes were paired in meiosis, they resemble a “parachute”. The Xy_p_ parachute system is considered the most common form of sex determination system in Coccinellidae family as well as in other families of Coleopteran insects (Smith and Virkki 1978, De Julio et al. 2010). However in some groups the Y chromosome has disappeared and the chromosomal system of sex determination changes to the X0 system (Angus et al. 2015).

Association of heterochromatic segments of all chromosome complement during early meiotic stages forming a single chromocenter has been described in *Epilachna vigintioctopunctata* Fabricius, 1775 (Bose 1948) and in *Epilachna paenulata* Germar, 1824 (Drets et al. 1983). This association has not been found in *Henosepilachna argus* (Figure 2) or any other species of Epilachnini, with the exception of the two previously commented species. However, associations of heterochromatic segments of non-homologous chromosomes in chromocenters have been described in others insects such as in Triatominae (Hemiptera) (Pita et al. 2014).

**Figure 3.**
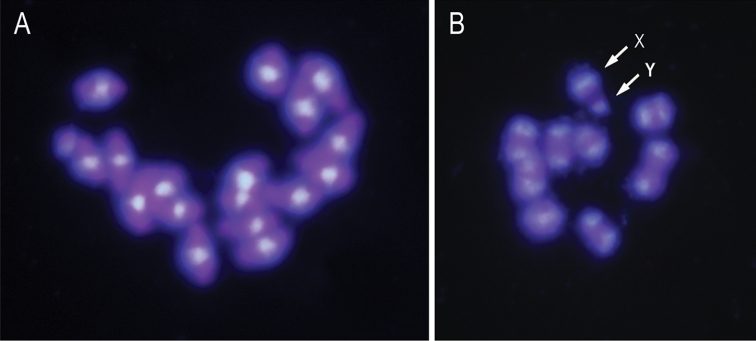
DAPI staining of mitotic metaphase (**A**) and meiotic metaphase I (**B**). The arrows indicate the sex chromosomes (X and y).

DAPI staining of mitotic chromosomes displayed similar results that C-banding technique with the pericentromeric chromosome regions intensely stained (Figure 3). Equally in meiotic metaphase I bivalents the pericentromeric regions were intensely stained. DAPI staining coincident with C-banding heterochromatic pericentromeric regions has also been found in some Coleoptera, suggesting that these heterochromatic regions are rich in AT base pairs although in other Coleoptera the DAPI staining of chromosomes did not reveal any positive signal (Karagyan et al. 2012, Da Silva et al. 2015 and references therein). This varied and different banding pattern could be due to the structure and composition of insect heterochromatin, especially in relation to the heterochromatin associated proteins (Lorite et al. 1996b).

**Figure 4.**
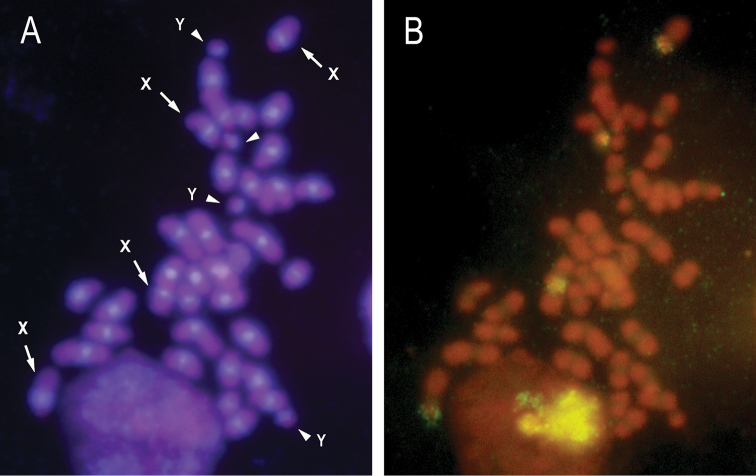
(**A**) Four mitotic metaphase plates stained with DAPI, the X chromosomes were showed by arrows, and y chromosomes were showed by the arrowhead. (**B**) FISH using rDNA as probe. Positive hybridization signals on the short arm of X chromosomes were showed.

The FISH technique using rDNA showed a positive hybridization signal on the short arm of X chromosome (Figure 4). The localization of the nucleolar organizer region (NOR) is unknown in other species of Epilachnini. At this moment the chromosome location of the NOR in Coccinellidae is only known in two species, *Olla v-nigrum* Mulsant, 1866 and *Cycloneda sanguinea* Linnaeus, 1763 (Maffei et al. 2001, 2004) by Ag-NOR banding and FISH. The NOR location in both species is variable. In the first one the NOR region appears on the sex chromosomes (Maffei et al. 2001), nevertheless in the second one the NOR region appears on one pair of autosomes (Maffei et al. 2004). The variable location of the NORs has been observed in other Coleopteran families as Carabidae, Melolonthidae, Tenebrionidae or Scarabaeidae that show that rDNA sequences are located on the sex chromosomes, autosomes or both depending on the species (Oliveira et al. 2012, Arcanjo et al. 2013).

**Figure 5.**
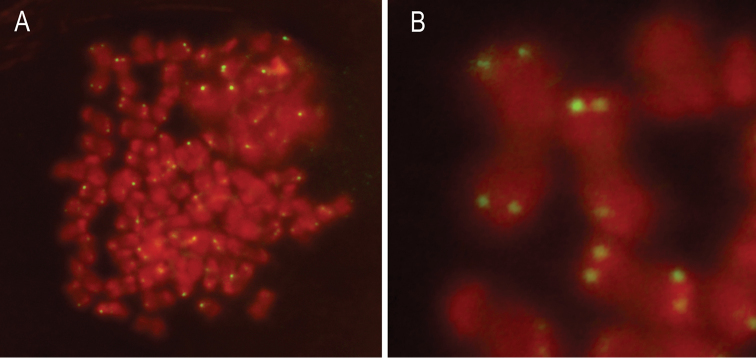
FISH using (TTAGG)n as probe on mitotic chromosomes (**A**) and selected chromosomes (**B**).

FISH showed that the TTAGG motif is present in the telomeres of the chromosomes of *Henosepilachna argus* (Figure 5). The pentanucleic repetition TTAGG is considered the most common telomeric sequence in insects (Frydrychová et al. 2004). In spite of this, the DNA composition of coleopteran telomeres is very variable. Frydrychová and Marec (2002) studied the occurrence of (TTAGG)n repeats in the telomeres of 12 species of beetles, which represent the major lineages of the phylogenetic tree of the Coleoptera order. Furthermore, the presence or absence of (TTAGG)n repeats was independent of the phylogenetic relationships. For example, in the suborder Polyphaga six species showed positive results to TTAGG probe and three negative results. In addition in *Tribolium castaneum* Herbst, 1797 (Tenebrionidae) there has been a replacement from TTAGG repetition to TCAGG repetition (Richards et al. 2008). Mravinac et al. (2011) showed that the motif TCAGG is found in all the 19 examined species of three beetle families belonging to the superfamily Tenebrionoidea, whereas TTAGG the canonical telomeric motif of insects, is found in most but not in all of the remaining species covering four families, Cucujidae, Cerambycidae, Chrysomelidae and Curculionidae. The analysis of the genome of *Tribolium castaneum* also showed that multiple telomeres are formed by TCAGG repetitions interrupted by full-length and truncated non-LTR (Long Terminal Repeats) retrotransposons. The authors also suggested that this type of telomeres should be a “middle” stage between the typical telomeres like in *Apis mellifera* Linnaeus, 1761 (Hymenoptera) (Robertson and Gordon 2006) and telomeres which are exclusively formed by non-LTR as in *Drosophila* (Diptera) (review by Zhang and Rong 2012, among others).

Thus, in this paper classical and molecular cytogenetic techniques have been performed on chromosomes of *Henosepilachna argus*. This is the first study on rDNA localization in Epilachninae. Besides, it is the first study of telomeric sequences in Coccinellidae family. This molecular cytogenetic study, in addition to expanding the knowledge of this species, could be helpful in the future for solving of the problem of distinctiveness between both genera
